# Expert system design for vacant parking space location using automatic learning and artificial vision

**DOI:** 10.1007/s11042-022-12906-z

**Published:** 2022-04-26

**Authors:** Juan Manuel Carrera García, Joaquín Recas Piorno, María Guijarro Mata-García

**Affiliations:** UCM: Universidad Complutense de Madrid, Madrid, Spain

**Keywords:** Computer vision, Machine learning, Neural network, Parking lot assistance

## Abstract

Finding a free parking space nowadays is a recurring problem in increasingly crowded public parking lots. The present study offers a solution that is based on the analysis of zenith images using artificial vision and is capable of automatically analyzing both the available spaces in the parking lot and their real-time occupancy. In an initial phase, the presented system semi-automatically detects the available parking spaces by filtering, thresholding, and carrying out a process of extracting the contour and approximating to a polygon the parking spaces of an empty parking lot. Once the size and location of the parking spaces have been mapped, the system is capable of detecting not only the presence of a vehicle in a parking space, but also the area of the parking space occupied by it with an accuracy of 98.21% using Region-based Convolutional Neural Networks. This feature allows the system to specify the appropriate parking space for a new vehicle entering the parking lot based on its specific dimensions and the correct location of the cars parked in the spaces adjacent to the free space.

## Introduction

How much time do people waste looking for a parking space? Who has not felt stressed out by having to drive around looking for a place when already being late for an important appointment, a meeting or an exam? These are realities and problems that people face in cities with a high volume of traffic.

With regards to this problem of parking space search, a general categorization is provided in [19]:

- Space-driven systems, which are based on the analysis of individual parking spaces to determine their occupancy according to whether or not a vehicle is present within the space, and
- Car-driven systems, which are based on the detection of vehicles with a subsequent analysis of their location in parking spaces.

The different works related to the above categorization are detailed below.

## Related work

In the literature, two main methodologies can be found when detecting vehicles in a parking lot, depending on what is identified: space-driven systems, designed to detect if there is an object inside a specific parking space; and car-driven systems, in which the system is trained to identify vehicles in a given area and thus infer the occupancy of parking spaces.

In this section, a review of the literature will be carried out, citing the most representative authors in each of the techniques, as well as the new proposal presented in this research work, in which not only the presence of vehicles is detected, but also their size and position within the region of the parking spaces, thus allowing the detection of parking spots that cannot be used due to the presence of badly parked vehicles in the adjacent spaces.

### Space-driven systems

In this section, the most relevant works within the space-driven systems category are listed, together with a brief description of the techniques used.

Preliminary work on this subject was undertaken by [12], who filed a patent in which Support Vector Machines (SVMs) are used for color classification within a parking space to distinguish cars.

In [40], a system that uses machine learning to detect empty places is proposed. The system is based on four steps: first, a preprocessing phase for the detection of parking spaces; second, the extraction of the characteristics of the ground; third, the application of an SVM classifier for vehicle recognition; and fourth, the use of a Markov random field model to detect blocks of three adjacent spaces in order to avoid errors due to occlusions or shadows.

A methodology for parking space detection is presented in [33]. In this work, the authors calculate 2D homographies by applying computational algebra algorithms, and use the homographs to generate a pseudo-view that can be used to obtain the occupancy status of parking spaces. The authors apply feature extraction techniques to this view and, finally, an SVM classifier which has been trained with the texture vectors of each place under different lighting conditions is used to detect the state of occupancy.

True [35] proposes a color histogram classification of parking spaces and an algorithm for detecting certain characteristics of cars to establish the occupancy status. For the histogram classification, two different classification methods are proposed and compared, namely K-Nearest Neighbors (KNN) and SVM, with the latter producing better results. And for the detection of vehicle characteristics, the Harris corner detection algorithm is used in the sub-image of each space. The parking spaces have to be classified and labeled manually in the initial configuration of the system.

The method proposed in [17] addresses the problem of occlusions by using several cameras distributed in the parking lot. The algorithm introduced by the authors is based on three stages: (i) preprocessing of the image is performed to eliminate the shadows and correct the distortion, (ii) the optimal processing image is defined with the evaluation of the state of the space to eliminate occlusions, and (iii) the occupancy of the parking space is evaluated.

In [19], the authors model the parking lot as a structure with several surfaces, which is obtained by using a Bayesian Hierarchical Detection Framework (BHDF). To do so, they propose a three-layer scheme composed of: a first layer for observation of the scene, a second layer for labels (car, ground, or shadow), and a final layer for 3D scene modeling. The framework is improved again in [20] by adding an analysis of the content of each parking space, which is modeled as a rectangular 3D prism. Finally, in [21] a multiclass reinforcement method is included to integrate debut classifiers, and the system is tested under nocturnal conditions.

Chen et al. [10] address the problem of managing outdoor parking spaces by using several cameras. In this work, all the images obtained by the cameras are synchronized and merged using different transformations to obtain the final image. Occupancy detection is performed by modeling the color changes within each parking space. For problems caused by perspective and occlusions, geometric models (ellipses and grids) are proposed to represent the space of a place, and is also used in this work.

In [22], a system for detecting free parking spaces in two-layer pre-trained neural networks is proposed. The system uses two neural networks, one during the day and the other at night, in order to limit the lighting changes during the classification of the state of the places. The system was tested with a 24-hour recording of an outdoor parking lot, obtaining an accuracy of 99.9% for occupied spaces and 97.9% for free spaces.

An approach based on the analysis of characteristics in the image is proposed in [7]. The authors propose the use of two types of analysis: one static and the other dynamic. In the static analysis, an edge detection algorithm and a classification of the color histogram for each space are used to evaluate the changes with respect to the empty place. Both features are combined using machine learning algorithms to create a decision tree that evaluates the final occupancy status. The dynamic analysis detects movement within the parking lot and is used as a correction mechanism for the resulting decision in case of changes in the spaces.

In [3] authors propose a simple method of detection based on the preprocessing of the parking lot image. In the image of the empty parking lot, circles are placed on each of the places. The algorithm identifies these points using the image process to determine the occupancy of the space. In [4] a further step is taken by adding extensive documentation on the architecture, communications, software and hardware used for the implementation of a system for this functionality.

In [5], the authors propose the use of a trained SVM with two descriptors of visual texture to determine the occupancy status of parking spaces. The system is improved and tested in [14], in which the authors create a data set consisting of 695,899 images of parking spaces. The images of the parking spaces were extracted from a previous set of 12,417 images of parking lots in different climatic conditions. These images were labeled and segmented to identify each of the parking spaces that appeared in them.

An approach based on vehicle volume is used in [16]. The system models 3D parking spaces to determine the volume they occupy in the image depending on the perspective. Vehicles are detected by classifying image pixels with SVM classifiers. Finally, the volume of occupation of the place is determined on the basis of the existence of vehicles in it.

In [24], a system is presented that combines the analysis of the density of the edges and the density of the background and foreground pixels in each parking space, which is defined by four points forming parallelograms. The spaces are analyzed individually to determine their occupancy status.

[36] present a study in which four different features, namely color, angle, Gaussian gradient difference histograms and Haar features, are compared to obtain the images in combination with three classification algorithms: KNN, linear discriminant analysis and SVM. The best-performing system is based on the color and histogram characteristics of the difference in Gaussian gradients, together with an SVM classifier.

[34] propose the extraction of visual features of the image —Oriented Gradient Histograms (OGHs), Gabor and SURF (Speeded Up Robust Features) histograms— to train KNN and SVM algorithms based on previously labeled images. The study focuses on detecting the occupation of parking spaces at street level. Tests and measurements are performed combining different visual features with both algorithms. The best results are obtained using OGH as a visual feature and KNN for learning the system. In a subsequent study [25] proposed an approach based on the previous conclusions. A parking space detection system is presented that comprises the following stages: (i) subtraction of the background, (ii) location of objects, and (iii) visual recognition. The system uses a pre-trained KNN classifier with images annotated manually by the authors, and an external database of tagged images. The classifier is capable of differentiating between vehicles and pedestrians.

In the system proposed in [15] two techniques are used: (i) background subtraction using a mixture of Gaussians to detect and track vehicles, and (ii) the creation of a transience map to detect the parking and exit of vehicles. The basis of the system is the temporal analysis of the video frames to detect the variation in occupancy of the parking areas.

In [29], an algorithm based on an SVM classifier is presented. Two features are used in the algorithm, namely the mean local entropy and the standard deviation of the mean entropy of the subregions of each parking space. Both characteristics are extracted from the scatter histogram of the parking spaces.

[1] present a framework for detecting the occupation of parking spaces using a Convolutional Neural Network (CNN) and an SVM classifier in outdoor parking lots. The classifier is trained and tested with the characteristics learned by the CNN from a set of images (called PKLot), which include examples with different lighting and weather conditions. The system reports a detection accuracy of 99.7% for the PKLot data set and 96.7% for its own data set.

One of the more recent approaches is provided by [37]. It analyzes parking spaces in blocks of three to infer the status of the central space, thus avoiding occlusion problems. To achieve this, the authors use various CNN-based techniques: (i) an initial CNN for spatial transformations, based on the size and position of the vehicles within the parking spaces for the next phase, with the aim of reducing perspective distortion problems; (ii) a CNN based on a siamese architecture, trained on the characteristics of the parking space; and (iii) a two-class logistic regression model that uses the output of the previous CNN to infer the final status of the central parking space.

One of the problems detected in systems based on the space-oriented approach is that each parking space is analyzed separately to detect whether it is empty or occupied, which generates a dependency on the position of a vehicle within the space, i.e. if a vehicle is parked between two spaces, or occupies part of two, there is a problem in considering each space as an individual area.

### Car-driven systems

In order to try to solve this problem, car-driven approaches have been developed. One of the first examples for detecting cars inside parking spaces can be found in [38], in which the authors propose a system based on the extraction of surface textures and microstructures to detect variation changes in an image when a car is present.

In other studies, such as that of [18], the images of the parking spaces are compared with a reference image of the empty place to detect changes in the pixels, while in [2] a classifier based on Haar characteristics is used to detect whether a car is present in a parking space, and thus determine its occupancy status.

On the other hand, [11] proposes an object detection algorithm based on adaptive background models. In this work, the author masks the scene with a background image in order to obtain the parts that correspond to the objects and analyze them individually. For these objects, the density of the edge orientation histogram is analyzed to determine the location and final occupancy status of the place through a decision-making module.

The work of [26] present a system that addresses how to determine occupancy status and the solution to the main problems it presents. The problem of occlusions between vehicles is solved. To do so, a novel system is proposed that, instead of monitoring the vehicles parked in the space, identifies the moving vehicles by eliminating the rest of the information in the scene. The resulting vehicles are located with respect to their position in the parking spaces - inside, outside, or entering. In [27], the problems of lighting and shadow changes are solved by using an adaptive background subtraction algorithm, while the SURF algorithm is used to solve problems related to rotation and scale changes. Later, [28] present a multi-agent system dedicated to the analysis of parking in a city.

The system proposed by [39] consists of three phases designed to determine the occupancy of parking spaces. First, the foreground features are extracted to obtain the background by using border and color features, and the regions of interest that the spaces represent are defined. Then, for each new video frame, the image of the adjacent difference with respect to the previous frame is generated, and the region of interest corresponding to each parking space is evaluated. Finally, to determine of the occupancy status, a method for updating adaptive thresholds is proposed.

One of the first approaches to use Convolutional Neural Networks (CNNs) for the detection of vehicles within parking lots is proposed in [41]. The proposed algorithm is based on two stages: (i) a trained CNN with a sliding window is used to search for and identify vehicles, and (ii) a distribution matrix is defined to obtain the placement density of the vehicles, with this matrix being used to eliminate redundant windows and detect vehicle positions.

In [6], the authors propose a decentralized and efficient solution for parking space occupancy detection that is designed to be integrable into smart cameras. The solution is based on a CNN inspired by the AlexNet architecture, as described in [23]. The authors reduce the size of AlexNet, obtaining an architecture that is 3 times smaller without any loss in detection performance. This is mainly possible because the original network, namely AlexNet, is capable of detecting 1000 different classes, and the authors simplify detection to two classes — empty and busy — to determine the state of occupancy.

In [31], a multi-camera system is proposed that performs the parallel processing of each image followed by the combination of the results. The algorithm is based on three stages: (i) the cars are detected in the image with the use of an R-CNN (Region-based Convolutional Neural Network), (ii) homographies and perspective corrections are applied to take into account the volume of the objects, and (iii) the vehicles are mapped onto the parking spaces to detect the occupancy status. The system was tested in the Pittsburgh International Airport parking lot by using the existing cameras.

### New approach

One of the main advantages of the new strategy presented in this paper, compared to previous works, is the use of a vehicle-oriented approach to detect the cars present in the parking lot. In this work, instead of using a binary approach like the solutions presented in the literature, in which a space is marked as empty or occupied, the percentage of free space (area) is obtained in each parking space. With this information, it is possible to provide information to the parking lot users about the specific spaces that a certain vehicle can use according to its characteristics and the status of the parking lot. In this way, the knowledge of the state of the space allocation is taken a step further, achieving the best possible optimisation of the parking space. If, for example, there is a badly parked vehicle invading part of an adjacent space, that space can only be used to park small vehicles or motorcycles, but certainly not large vehicles.

Another advantage of the solution presented in this work compared to previous works resides in the use of instance segmentation using an R-CNN, instead of object detection, as a method for vehicle detection. This more advanced technique allows the system to detect and generate the contours of the vehicles in real-time for their subsequent location in the parking lot. The advantage of this method with respect to systems based on image segmentation is the elimination of the problems produced by changes in lighting conditions, weather, and those caused by the shadows that occur in them. In comparison with the space-driven systems, which train classifiers that infer whether a space is empty or occupied, the advantage here is the improvement of the data necessary for their training. Instead of needing images of parking lots to train the system, the process is generalized by using images of vehicles to train the neural network.

Another feature of the system presented in this work provides something that few systems in the literature contemplate, namely the automatic detection and mapping of the coordinates of the parking spaces to obtain a computer model that can be used by the system. Many authors perform this task manually during the setup phase, or even ignore it. Performing the task in an automated way is a clear advantage, especially in large parking lots, where the manual labeling of spaces can require a great effort, and this makes the system presented in this paper more scalable.

Finally, the most significant advantage is the implementation of a dedicated search algorithm to find the optimal parking space for a vehicle entering the parking lot according to its characteristics and the parking lot status. This has not been considered in any of the existing publications to date, and in the present work it is considered that it is a great help for the user’s experience with the system, since it is able to indicate the location of a suitable parking space for the vehicle when entering the parking lot, thus avoiding the need to spend time looking for a free space to park.

## Methodology

The proposed system is based on the creation of a system focused on the management of parking space occupation in outdoor environments through image analysis. For this purpose, the system is provided with intelligence by means of artificial vision and automatic learning -using neural networks-, thus allowing it to process the images with which to recognize the environment, identify the lines that define the parking spaces, recognize the vehicles inside the spaces and evaluate the final state of occupancy.

### Overview

**Fig. 1:**
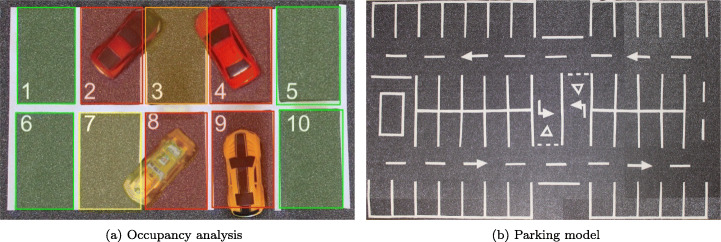
Parking model

An important improvement with respect to the current systems is the determination of the occupancy status based on the area occupied by the vehicles present in the parking spaces. In this way, it is possible to solve the problems of false positives that can be caused by vehicles that are badly parked in the spaces, taking the knowledge of the state of the parking lot and the optimization of the distribution of the space a step further. Figure 1a illustrates a problem that arises from the poor placement of vehicles in parking spaces. With the determination of the state of the binary space - empty or occupied -, both space number 3 and number 7 would be marked as occupied because there are vehicles in them. However, by using an area-based approach, these spaces can be used to park a motorcycle, for example, or even a small vehicle when there are no other options in the parking lot. In addition, the status of each parking space is identified with different levels, according to the area occupied in each, via the colors: green, yellow, orange and red.

The system goes beyond the detection of the occupancy status of parking spaces by being able to detect and recognize vehicles, differentiating them from the rest of the elements that can coexist within a parking lot, and also determine the area occupied by these vehicles in each parking space. To detect changes in the state of the parking spaces and update the general state, the system includes motion detection algorithms to know when there are changes within the parking lot.

Since the system is based on image analysis, and to cover as many cases as possible, a scale model has been created to simulate different vehicle placement conditions, and scenarios with changing lighting conditions.

Furthermore, since the topology of parking lots and vehicles in a simulated environment is very similar to that in a real one, the use of a model makes it possible to carry out an initial training stage with which the network is able to recognize these common features with a high success rate. This neural network will be improved afterwards with further training on a reduced set of images in real environments. Otherwise, the training set of real images would have to be much larger, with the consequent difficulties involved in their generation.

As shown in Fig. 1b, the model consists of: (i) an entrance and exit for vehicles, (ii) six blocks of parking spaces with different topologies, (iii) a space enabled for the detection of the incoming vehicle where the size of the vehicle accessing the parking lot for its subsequent location will be determined, and (iv) two pedestrian access points that may be, for example, the different entrances of a shopping centre.

To present the system, the origin of each of the modules in the implementation is described: (i) the automatic detection and location of parking spaces is one of the characteristics that were avoided in previous works, but needed in a real system; (ii) the detection of vehicles by neural networks capable of recognizing and generating the contours of vehicles on the scene to determine the state based on the area occupied by each one; and (iii) measurements on the scene to determine the real size of objects of interest.

The final objective of these modules is to obtain a computational representation of the parking lot and the objects located within it. Each one of the modules provides the representation of the physical entities contained in the images as final objects that are handled in the system for the management of the parking lot’s occupancy.

### Parking space detection

**Fig. 2:**
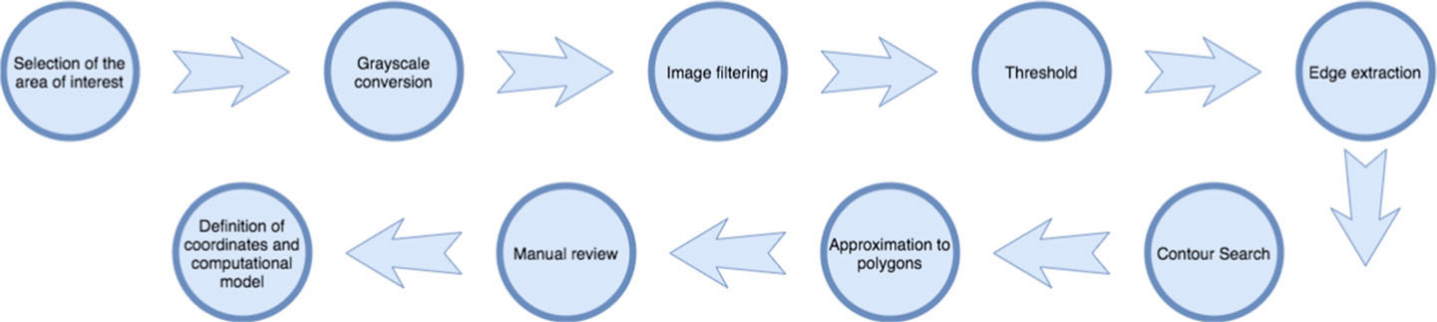
Stages for parking space detection

To detect the parking spaces, the image of the empty parking space is processed, the lines that define each of the spaces are detected and a computer map of them is obtained. Each parking space is defined in the system by four coordinates that form a quadrilateral. Figure 2 shows the stages followed for the segmentation of the parking spaces in order to obtain figures based on coordinates, which are computationally manageable by the system.

Since there are many lines in a car park in addition to those corresponding to the spaces, for example those of signs, lane markers, pedestrian crossings, etc., the spaces are analyzed in blocks or regions of interest. A block is formed of a set of adjacent and neighboring squares defined by an expert. Figure 1b shows the existing regions of interest in the designed parking model.

#### Selection and filtering of the region of interest

By selecting the Regions of Interest (RoIs) in an image, each of the parking spaces is analyzed in isolation. In this way, each RoI is intended to contain only the lines corresponding to the parking spaces in a block to improve automatic detection.

Filtering an image is a process used to highlight, enhance, smooth, or suppress certain characteristics of the image. Filters are used both to detect elements or edges and to remove elements such as noise in an image. In both cases, sharp changes produced by high-frequency components are detected.

**Fig. 3:**
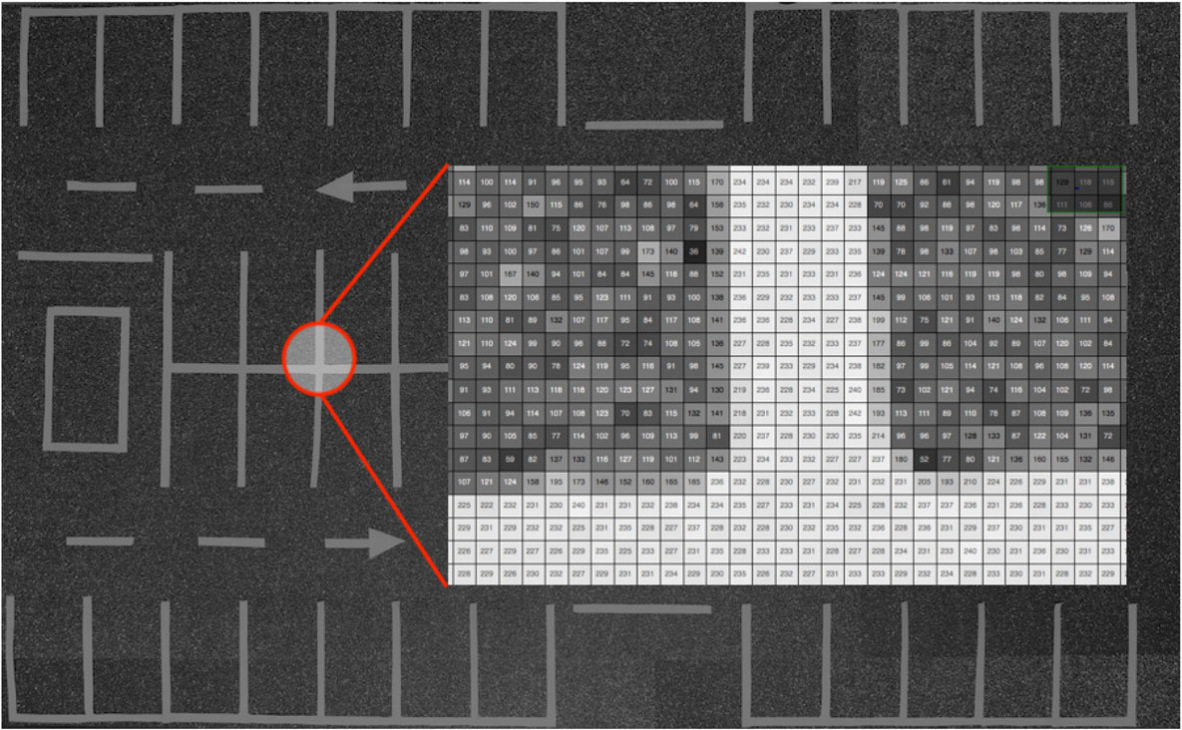
Gray level values of a portion of the empty parking lot image

Apparently, the original image of the parking lot -Fig. 1b- does not show any noise. However, if the image is analyzed in detail -Fig. 3-, thus showing the gray values of a sub-matrix of the image, the changes in level (noise) can be seen. For this reason, the system uses the first filtering to reduce the noise that the image on which the detection is made may contain.

The filtering process consists of transforming the original image by applying a convolution matrix - core or kernel - to each pixel, affecting the central and adjacent pixels. These matrices are square and usually have dimensions of 3x3, 5x5, etc.

In this work three filters, namely Gaussian, median and bilateral, were analyzed because these are the most commonly used in the literature. This was done to check the result produced by each one and to evaluate their suitability for the system. Of the three algorithms the median filter is the one that produces the best softening of the background, thus highlighting the lines of the parking spaces.

**Fig. 4:**
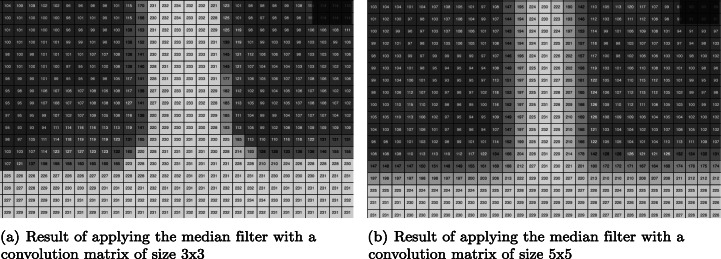
Size comparison of convolution matrices used with the?median filter for the segmentation of parking spaces

After analysing the behaviour of the median filter with various convolution matrix sizes, the optimal convolution matrix size is determined to be 5x5, thus obtaining the best results, smoothing and removing more noise in the background areas, and preserving the edges corresponding to the lines. Figure 4 shows a comparison of the results obtained by applying 3x3 (4a) and 5x5 (4b) convolution matrices, while larger window sizes generate worse results in the smoothing and denoising process.

#### Image threshold

Threshold value methods are algorithms that allow us to segment images for object extraction. The threshold is based on the determination of a threshold value that is used to determine whether the pixel analyzed is part of the background or the object of interest. Since the threshold value is set to a gray level, the image to be segmented must be in grayscale.

**Fig. 5:**
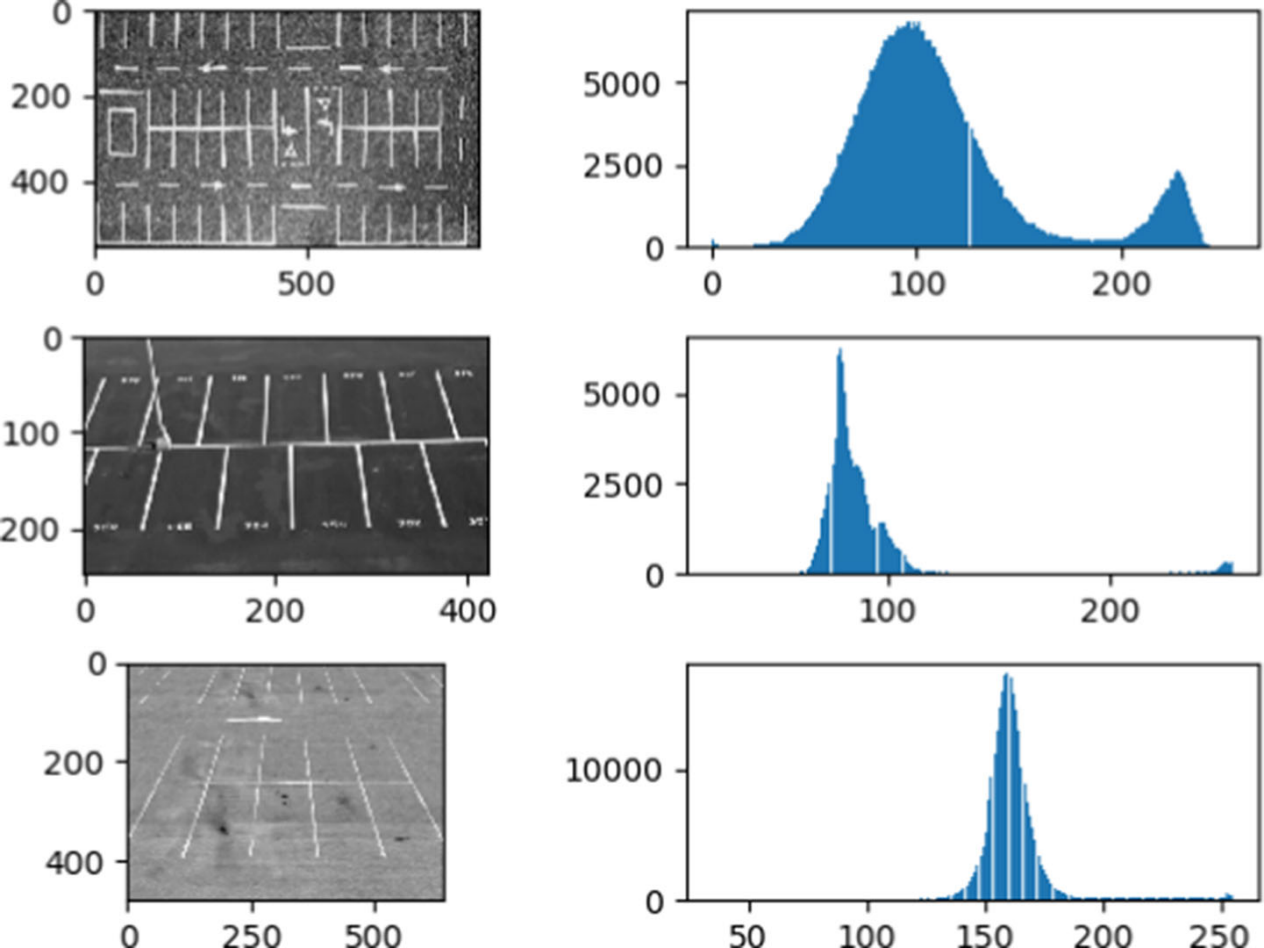
Histograms of gray levels in empty parking lots and Otsu’s threshold

To determine the optimal threshold method for the search of parking spaces in the system, the histograms of several images corresponding to empty parking spaces are analyzed. Figure 5 shows the histograms of three images in which it can be seen that, to a greater or lesser extent, the distributions are bimodal. In general, empty parking spaces have this type of distribution, in which the values on the left correspond to the background (ground), and those on the right to the objects to be segmented (parking lines). Therefore, it can be concluded that, by presenting this type of distribution, the optimal method for finding the threshold value is [32]. This method requires that the histogram of the image presents a bimodal distribution to automatically calculate the overall threshold value and segment the image based on this value.

#### Edge extraction

Edge extraction is used to identify abrupt changes in intensity between adjacent pixels. These changes correspond to the line contours of the parking spaces.

The histogram with the intensity profile of the lighting corresponding to empty parking lot images in grayscale is shown in Fig. 5. The histogram shows that the edges - changes in the graph - are presented as a progressive change in the intensity level instead of sudden changes.

The system uses the Canny algorithm [8] because, in addition to detecting edges in the image, it is able to detect and differentiate contours. This feature is perfect for parking space detection as it favors the extraction of parking spaces as contours in the image.

**Fig. 6:**
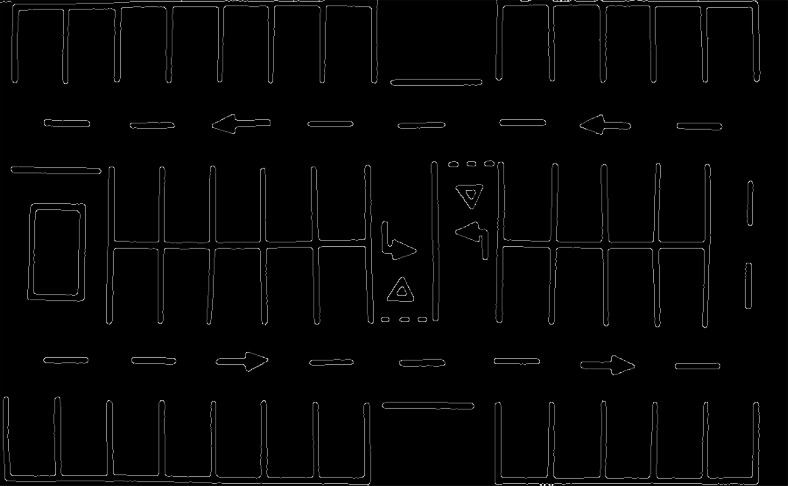
Edge extraction using the Canny algorithm

With the application of the algorithm, the lines of the parking spaces are segmented so that they are defined by their contour, eliminating the interior color of the parking spaces. This thinning and contouring makes the definition of the coordinates of the parking spaces easier in later phases of the algorithm, thus improving automatic detection. Figure 6 shows the result generated by applying the Canny algorithm to the system.

Now that the image has been segmented, the noise has been eliminated and the contours of all the lines have been defined, the methods used to obtain the final coordinates of each of the parking spaces can be explained.

#### Contour detection and polygon approximation

**Fig. 7:**
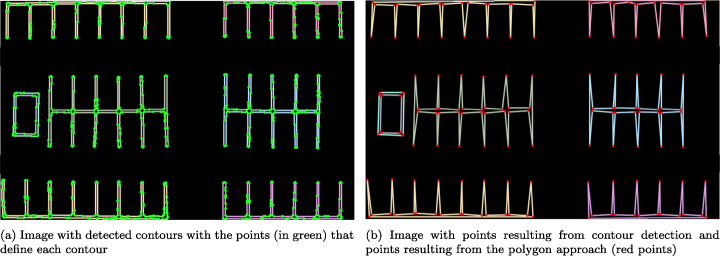
Detection and drawing of contours with the points resulting from the algorithm

Contour detection is used in the system to obtain the final coordinates of the parking spaces. By using this method, each parking lot block is identified and differentiated as a contour that is treated independently from the other parking spaces and image elements. Figure 7a shows the different contours detected in the parking lot image.

If you analyze Fig. 7a in more detail, you will see that even when using the polygon approximation method, which generates the least number of points possible, the lines are not completely straight and have small variations that cause each line to be defined internally by many points. Therefore, to reduce the number of points and find the simplest representation of the parking lot to define the coordinates of the spaces, a polygon approximation is implemented using the Ramer-Douglas-Peucker algorithm [30].

Figure 7b shows the final result of the contours drawn on the basis of the points obtained by applying the Ramer-Douglas-Peucker algorithm. It can be observed that the number of points is reduced while preserving those necessary to define the coordinates of the contour.

However, there are still redundant points in the crossing areas between the vertical and horizontal lines in the parking blocks for the detection of parking spaces. In Fig. 7b this problem can be seen in some crossings that have up to 4 different points in the central separation of a block. To minimize the number of points, all those that form a contour (parking block) are processed by grouping the points where their Euclidean distance [13] is less than a threshold value, and calculating the midpoint of the group, thus generating a single coordinate at the vertical and horizontal line crossings. The ends of the vertical lines of the parking blocks are correctly reduced by using a single point at each end.

**Fig. 8:**
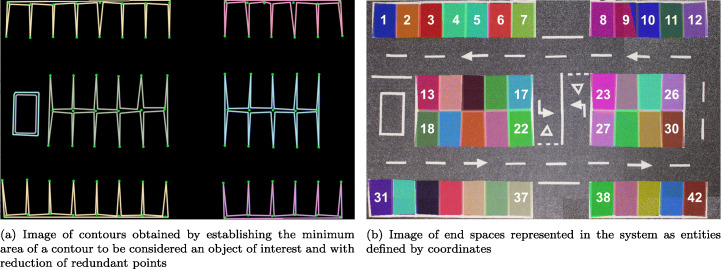
Image with final contours and definition on the original parking lot

It should be noted that both the lines of the parking spaces and the other road markings are detected. This case is covered in the system by processing each of the parking blocks separately, selecting the RoI of the parking spaces to be segmented and leaving out the marks external to the processed block. However, to mitigate a possible selection error, an algorithm is implemented that takes into account the minimum area that the detected contours must have to be considered as parking blocks or objects of interest. The result of these optimizations is shown in Fig. 8a.

Figure 8b shows the final result, with the drawing of the entities detected as spaces in the system.

#### Manual adjustment and computer model of the parking spaces

Since the detection of parking spaces is a critical step, as a faulty definition would make the detection of occupancy incorrect, a final stage is proposed that would only be necessary in the configuration phase of the system, and in which a manual check is carried out of the coordinates of the parking spaces obtained. This corrects possible errors such as that shown in Fig. 8b, in the top row in spaces 9 and 10, which was caused by obtaining an incorrect coordinate.

The result of the automatic detection process described and the manual check and correction, if necessary, generate the coordinates of all the parking spaces in the system. In this final step, the coordinates are grouped and the objects corresponding to each parking space with which the system works are generated.

### Vehicle detection in the parking lot

A task as simple for a human as identifying the vehicles in an image is a very complicated process for a machine. Machines *see* an image as a set of bits, and by abstracting at the highest level, it can be said that they are able to *see* the image as a matrix, in which each element represents a color, but it is not easy for them to say how many vehicles there are or where they are.

With regards to the complexity of detection and the aims of the system, namely to i) detect and identify all the vehicles in an image, and ii) calculate with great precision the surface occupied by each vehicle, these have been addressed in previous research and work carried out by the same authors [9].

The characteristics of the final system obtained in the above work, with the objective of providing the system used here with the capacity to see, detect, delimit and measure the area of the vehicles seen from a zenithal position in an image, discarding the rest of the objects such as pedestrians or shopping carts, are summarized below.

The two basic techniques used in previous work are: neural networks, specifically region-based convolutional neural networks (R-CNNs), which are a type of CNN focused on object detection; and object detection techniques, using instance segmentation. Convolutional neural networks offer a set of powerful algorithms that allow a computer to solve problems that are trivial for a human, but which have a very high level of complexity for a computer, providing the system with the capacity for sensory perception. Instance segmentation identifies the category of each pixel in an image by differentiating between pixels of the same category that belong to different objects of the same class, predicting the mask of the identified objects of interest, thus obtaining the contour of the vehicles present in an image. Both features converge in a type of CNN, namely region-based convolutional neural networks, which are the backbone of vehicle detection.

Region-based convolutional neural networks (R-CNNs) are a type of CNN focused on object detection. R-CNNs divide the image into a set of regions of interest, based on a selective search, and then process each of the regions using a CNN to categorize the objects and frame them. Specifically, the method known as Mask R-CNN is used, and implemented with the following techniques:

- A backbone neural network based on ResNet101 and FPN (Feature Pyramid Network) architectures. The ResNet101 neural network is used as a multi-layered feature extractor, in which the first layers detect low-level features, such as edges and corners, and the following layers detect high-level features, defining the object’s category. As an improvement upon the ResNet101 extraction pyramid, FPN is included by adding a second pyramid that takes the high-level features of the ResNet101 layers and passes them to the lower layers. This allows features at all levels to access lower- and higher-level features.
- A lightweight neural network called RPN (Proposed Region Network) to determine proposed regions by scanning the image using a sliding window to find areas with objects.
- A stage of analysis of the regions of interest (RoI) proposed by the RPN that generates two outputs for each RoI if it detects an object: (i) the class of the object within the region, and (ii) a bounding box refinement of the object to further refine the location.
- A final stage that generates the segmentation masks of each detected object based on the RoI of the previous stage.

The final result obtained in this previous work is a system capable of identifying and masking the contours of the vehicles present in an image with an accuracy of 97.98%. This is the method that has been used for vehicle detection in this work.

### Measurement on the scene

On-scene measurement is the process by which the system is able to know the size of the different objects that coexist in the parking lot. These measurements are necessary to obtain: (i) the area of the parking spaces, (ii) the area of the parked vehicles, and (iii) the area of the vehicles entering the parking lot. Thanks to (i) and (ii) the system is able to determine the area occupied in each parking space, and through (iii) the system obtains the necessary information to later determine the optimum space for the incoming vehicle. Figure 1b shows the area dedicated to vehicle detection, in which the size of the vehicles entering the parking lot is obtained for their subsequent location. These measurements and the determination of the state of a parking space based on the occupied area are the reasons why the use of the zenithal plane is considered. The use of the zenithal plane in the system makes it possible to (i) reduce to a minimum the distortions caused by perspective, (ii) eliminate the occlusions produced between vehicles, and (iii) consider the parking lot as a 2-dimensional model.

### Occupancy detection algorithm

At this point, the system has the basic tools and algorithms needed to (i) detect parking spaces, (ii) detect vehicles in the parking lot, and (iii) take measurements on the parking lot. By using all these, the algorithm that is capable of detecting the status and area occupied in each of the parking spaces can be defined, making it possible to determine the general occupancy status of the parking lot.

Before defining the algorithm, and since the aim is not to perform a binary detection -empty or occupied- of the status of the parking spaces, but to calculate the area occupied in each one, it is clear that the algorithm must take into account: (i) the area occupied by each vehicle, and (ii) the **position of the vehicle** within each space.

**Fig. 9:**
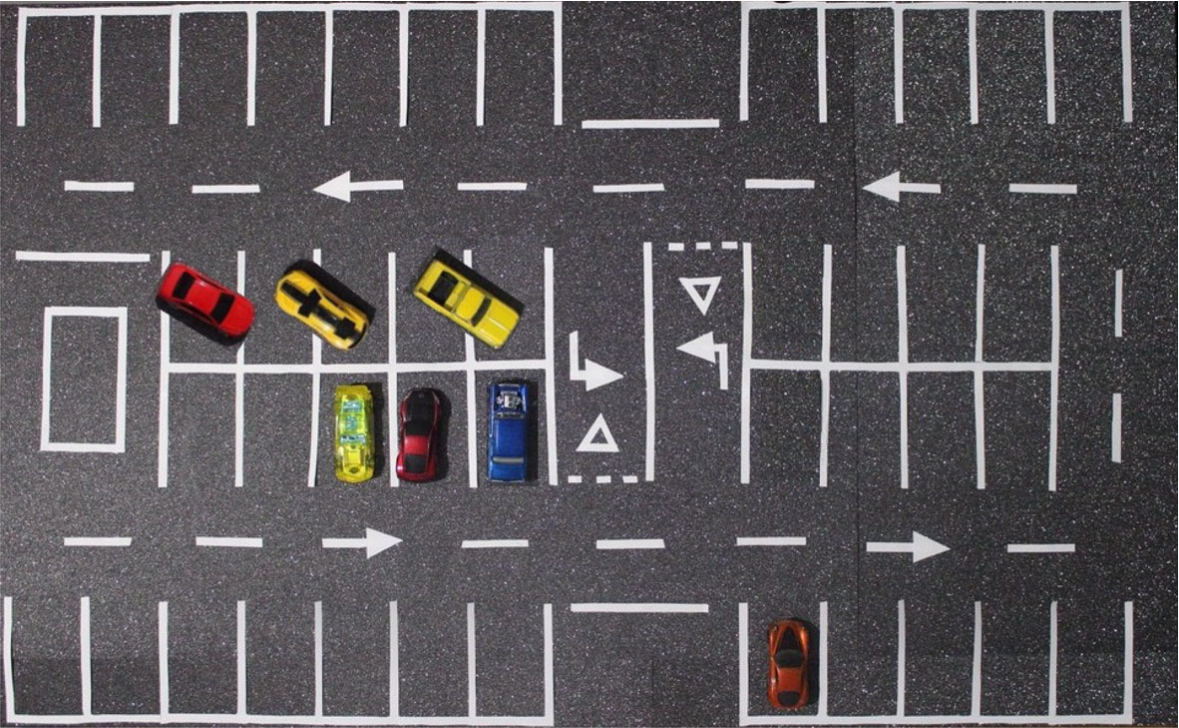
Position of the vehicles in the parking spaces

The importance of taking into account the position of the vehicles is illustrated in Fig. 9. It can be seen that in the upper row three vehicles occupy five spaces, while in the lower row the same vehicles occupy three spaces. While it is true that these are extreme cases, it is important to take into account these phenomena to prevent the algorithm from returning false positives in which a space is detected as free, but is actually occupied by the bad placement of the vehicles in the adjoining spaces.

When the algorithm is used, the system knows the location of each parking space and is able to detect vehicles and establish measurements within the parking lot. Therefore, the remaining steps are: (i) to detect, for each vehicle, the spaces it intersects, and (ii) to normalize the area of the vehicle on each of the spaces it intersects.

**Fig. 10:**
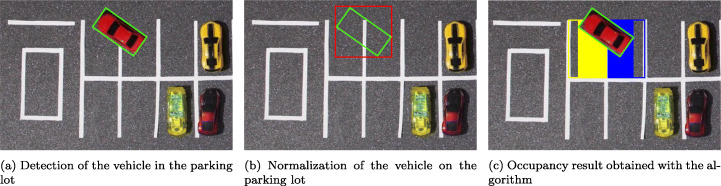
Graphic analysis of the detection algorithm for the occupied area

Figure 10 shows a graphical analysis of the steps followed by the algorithm (1) to detect the occupancy of parking spaces. The steps to obtain the final detection are:

1. Vehicles located in parking spaces are detected, approximating their shape to that of a quadrilateral (Fig. 10, Algorithm 1 line 1).
2. The area of the detected vehicle is normalized by creating a new quadrilateral that is parallel to the parking lines (Fig. 10b, Algorithm 1 line 3);
3. Based on the normalized quadrilateral, the parking spaces with which it intersects are located (Fig. 10c, Algorithm 1 line 4);
4. And finally, their percentage of occupation is obtained (Algorithm 1 line 7);

On the basis of these premises, the algorithm —1— consists of the following steps: 
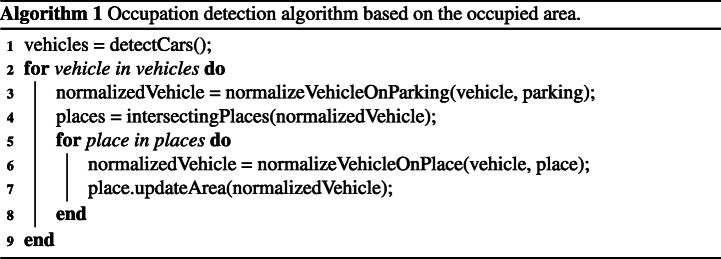


## Results and discussion

Since the main purpose of this work is to develop and analyze a proof of concept of a robust and valid system for determining the occupancy of parking lots based on the occupied area, a main set (Set A) of images obtained from the model is used. In this way, multiple different scenarios can be modelled, with vehicles in different positions - well and badly parked - and with changing lighting conditions. This generates a dataset containing the same situations that could be obtained by recording a real scenario over several days. This model dataset has been used during the COVID-19 (Coronavirus Disease 2019) pandemic shutdown to train the network, obtaining the results presented.

Nevertheless, this work would be meaningless if it did not serve as a basis for a functional system in real environments, so a secondary set (Set B) is used with images obtained from a real parking lot. This set seeks to evaluate the behavior of the system in real parking lots, in order to be able to affirm that the proof of concept of this work is valid and can be extrapolated to these environments.

For the analysis of the system, the different parts or modules that compose it are evaluated. First, the precision in the detection of parking spaces and vehicles is evaluated, and then the precision of the general system is assessed through the results of the occupation algorithm.

To determine the accuracy of the measurements obtained from the images of the parking lot: (1) the value of the areas of the objects of interest - vehicles and parking spaces - is obtained manually, (2) through the system the areas of these same elements are computed, (3) both measurements are compared obtaining the percentage of success of each one, and (4) the average value of all the measurements obtained is calculated.

Thus, the first stage in the analysis involves the detection of the parking spaces with which the system obtains a computer representation of the position of each parking space. This is something that had not been done in previous works and that in a real system would be fundamental to optimize its configuration. The accuracy obtained in the determination of the area of the parking spaces is 95.90%. However, since this detection is only performed once in the configuration phase of the system, and it is essential to obtain a completely correct computer map of the parking area, a manual correction stage is enabled. This is carried out by an expert, and enables small detection errors to be corrected.

Once the parking lot is mapped in the system, the accuracy of the system in determining the area of the vehicles is evaluated. This is the critical part of the system, since it is the only one with a total dependence on the environment, due to variable conditions that are external to the system such as luminosity or the topology of the objects of interest. In the determination of parking spaces, this dependence is mitigated with manual correction, and with regards to the occupation algorithm, it only works with abstractions in the form of computer models of the parking lot and vehicles, which are generated in previous stages. Therefore, in addition to calculating the accuracy with the images of vehicles from Set A, which correspond to the model, images with vehicles from Set B are included to check the accuracy in real environments and that the proof of concept of the solution presented in this paper is valid. The final accuracy obtained with the vehicles in the model (Set A) is 98.63%, and with the real vehicles (Set B) it is 97.98%.

**Fig. 11:**
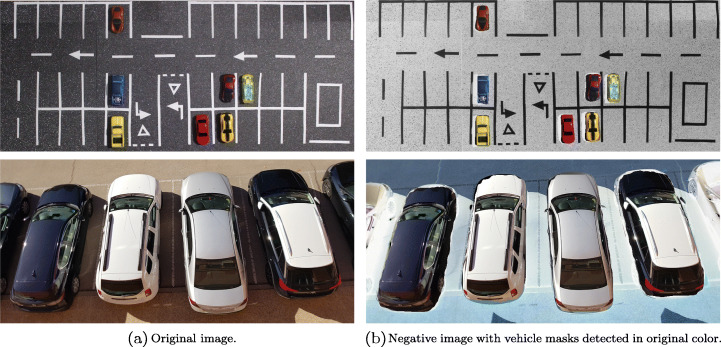
Vehicle detection in a parking lot

Figure 11 shows examples of the detection of vehicles from Set A and B, in the top and bottom row, respectively. Figure 11a shows the original images, and Fig. 11b shows the result in which a negative effect is applied to all the pixels of the original image, except those in which vehicles have been detected, maintaining the color of the original pixel in them.

At this point, the system has, on the one hand, a representation of the parking lot generated in the configuration stage and, on the other, the position and points that define the contour of the existing vehicles in the parking lot. In other words, there is a separate computer representation of the elements of interest - places and vehicles - necessary to determine the final occupancy of the parking lot. Both elements are used together in the parking lot occupancy detection stage. In this stage, the occupation detection algorithm is applied to determine the free and occupied area of each of the parking spaces, thus obtaining the final occupancy of the parking lot. The process followed by the algorithm to determine the area occupied by each vehicle is defined in point Section 3.5 and explained graphically in Fig. 10.

As can be seen in table 5 of [34], previous literature uses a large number of images to determine the accuracy of the systems, but many of these images are obtained from video frames of a car park, which makes many of them similar to each other. For this study, a carefully selected dataset (dataset A) has been prepared, with images of vehicles placed in different positions (well and badly parked) and with variations in the lighting conditions; in order to contemplate all possible casuistry and to be able to cover all the problems that could be found in a car park. The resulting dataset contains a total of 226 vehicles and 840 parking spaces that are analysed and the results presented by comparing the value obtained manually for the area occupied by the vehicles within the parking lot with that obtained by the system.

In order to establish a comparison between previous work and the new approach presented in this paper, the accuracy of the system under a binary approach is analysed. To make the correspondence between the area-based states -“*free*”, “*not very occupied*”, “*very occupied*”, and “*occupied*”- and the binary approach -free and occupied- it is established that vacancies whose occupancy is less than 10% should be detected as free and when occupancy is equal or higher than 90% should be detected as occupied.

**Fig. 12:**
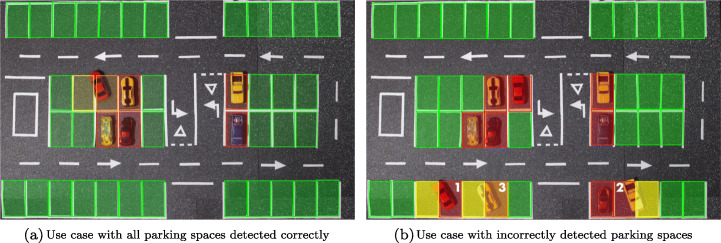
Occupancy analysis

Table 1 shows the results of 20 different scenarios in which the number of vehicles present in the parking lot has been modified (column 1), as well as their location, see as an example Figure 12a, corresponding to 6 vehicles with 1 of them badly parked, and Fig. 12b, corresponding to 11 vehicles in which 3 of them are badly parked, for a total of 42 spaces (see analysis later). For this table, the results of the binary analysis can be seen in the “*Binary precision*” section, in which 3 columns can be distinguished: the first corresponding to the number of free places compared to those detected as free; the second to the occupied spots (real/estimated); and the third the precision of the system. In this section of the table it can be seen how the system reaches 100% accuracy in most of the scenarios, with an average of 99.05%, and the one that offers the worst results (95.24%) is that corresponding to the presence of 16 vehicles. This example presents worse results than the rest because one of the vehicles is particularly poorly parked, as it is exactly between two spaces, and the binary detection does not detect it, marking the two spaces as empty. While it is true that this result may not be entirely fair because high resolution images have been used in this work, in which the objects are well defined and there is no loss of information, it can be used as a reference between previous approaches and the current work.

**Table 1:** Comparison of the real state/inferred state of the parking spaces and accuracy obtained in each scenario

	Binary precision			Area based precision				
Vehicles	Free	Occupied	Accuracy (%)	Free	Not very	Very	Occupied	Accuracy (%)
					occupied	occupied		
1	41/41	1/1	100.00%	41/41	0/0	0/0	1/1	97.96%
2	40/39	2/3	97.62%	39/39	1/1	1/1	1/1	98.19%
4	38/38	4/4	100.00%	37/38	1/0	1/1	3/3	97.55%
5	37/36	5/6	97.62%	35/36	2/1	2/1	3/4	96.91%
6	36/36	42/42	100.00%	35/35	1/1	1/1	5/5	98.31%
7	35/35	7/7	100.00%	34/34	1/1	1/1	6/6	98.46%
8	34/34	8/8	100.00%	33/33	1/1	1/1	7/7	98.90%
9	33/33	9/9	100.00%	33/33	0/0	0/0	9/9	98.80%
10	32/31	10/11	97.62%	29/29	3/3	3/1	7/9	98.17%
11	31/31	11/11	100.00%	29/29	2/2	2/2	9/9	98.40%
12	30/30	12/12	100.00%	27/27	3/3	3/3	9/9	98.98%
12	30/29	12/13	97.62%	29/30	1/0	1/1	11/11	97.84%
14	28/27	14/15	97.62%	27/27	1/1	1/0	13/14	97.58%
15	27/27	15/15	100.00%	26/26	1/1	0/0	15/15	98.92%
16	26/28	16/14	95.24%	22/23	4/3	4/3	12/13	97.62%
17	25/25	17/17	100.00%	22/22	3/3	3/0	14/17	97.94%
18	24/24	18/18	100.00%	22/22	2/2	2/2	16/16	98.51%
19	23/23	19/19	100.00%	22/22	1/1	1/1	18/18	98.89%
20	22/22	20/20	100.00%	19/20	3/2	3/3	17/17	98.19%
20	22/21	20/21	97.62%	20/21	2/1	2/1	18/19	97.77%
Mean			99.05%					98.21%

In the second section of Table 1, “*Area based precision*”, the same information is presented, dividing the column corresponding to the occupied spaces into three subcategories: “*not very occupied*” (space occupation of less than 15%); “*very occupied*” (occupancy between 15% and 85%); and “*occupied*” (occupancy greater than 85%). For this case, it can be seen how the accuracy of the system is much more homogeneous, hovering around the average value of 98.21% in all cases. When the area based method is used, errors can be found in cases where the vehicles are poorly parked and the difference from one state to another is minimal, causing any minimal inaccuracy in the segmentation of the vehicle to generate an error. For example, in the scenario with 17 vehicles, 3 were poorly parked occupying an area of approximately 88% of the square. These spaces should have been detected as “*very occupied*”, and could be used for a very small vehicle, for example a motorcycle, but the algorithm detects them as “*occupied*”.

Figure 12 shows two examples corresponding to use cases included in the table for the precision analysis of the algorithm.

Figure 12a shows a use case with all the parking spaces detected correctly, which corresponds to the use case with six vehicles in the parking lot, five parked correctly and one incorrectly. The final result in this case is a parking lot with thirty-five (35) free spaces -green spaces-, one (1) space that is not very occupied -yellow space - and one (1) very occupied space -orange space-, occupied by the same vehicle that is parked incorrectly, and five (5) spaces that are completely occupied -red spaces-.

Figure 12b shows a use case with some parking spaces detected incorrectly, which corresponds to the use case with ten vehicles in the parking lot, seven parked correctly and three incorrectly. The three cars parked incorrectly in the spaces marked 1, 2 and 3 each occupy two spaces -one not very occupied and one very occupied—. The system’s detection is incorrect in spaces 1 and 2, as in these two spaces the detection should be the same as in space 3, namely one not very occupied and one very occupied, but in this case the very occupied spaces are detected as occupied. The final result in this case is a parking lot with twenty-nine (29) free spaces -green spaces-, three (3) not very occupied spaces -yellow spaces-, one (1) very occupied space -orange space- and nine (9) occupied spaces -red spaces-.

The final accuracy of the algorithm obtained with the average of all the tests, and therefore of the system, in the detection of the occupation of a parking lot based on the area of vehicles and spaces is 98.21%.

The execution time is calculated for each of the modelled scenarios. The Table 2 shows the minimum and maximum value, as well as the mean and standard deviation of the results. It can be seen that the proposed system takes on average 1.52s to process each of the images.

**Table 2:** Analysis of runtime in the modeled scenarios

T (s)	
avg ± std	1.526 ± 0.160
min	1.299
max	1.927

Based on these results, it can be concluded that the approach of a vehicle area-based detection system is valid. The system is able to obtain an accuracy of 98.21% in the simulated environment and tests in real environments show promising results with an accuracy of 97.98%.

For the implementation and application of the system in a real environment, a distributed architecture is proposed, composed of several computing nodes with different characteristics and that perform different system tasks in a modular way. The necessary computing nodes in the system would be the following:

- WiFi IP Cameras: these are the devices in charge of capturing the images on which the state of occupation of the places is evaluated. Each camera captures a set of parking spaces corresponding to one or more parking blocks. The use of WiFi cameras is proposed to avoid the installation of network cabling. For power, the cameras can be attached, for example, to light poles in the car park to have a power outlet and enough height to have a zenithal view of the area to be covered.
- Local Processing Nodes: connected to one or more IP cameras, depending on the computing capabilities, the nodes process the video images to detect parking lot occupancy and send the information to a Central Server. Unlike systems where images are processed on a central server, performing distributed processing improves system scalability by avoiding bottlenecks.
- Access control nodes: they are used to process images of incoming vehicles to detect their size and send the information to a Central Server. The server returns the optimal parking place based on the vehicle size.
- Central Server: responsible for centralizing the information by exchanging data with the rest of the nodes. The server must include: (i) the software that contains the business layer of the application, (ii) a database management system to store the system information and (iii) a data server used by the nodes for the data exchange. The server must provide an API with which the rest of the nodes interact to obtain and publish information from the system.

Communications between the different nodes of the system would be done through a WLAN network using the IEEE 802.11 standard. The information would be exchanged between the nodes of the system using the JSON format.

Nevertheless, the system has some limitations in the images needed, as it requires high quality, high altitude, zenithal photography, which rules out its use in indoor car parks. With respect to the execution time of 1.52s, it is sufficient for a car park environment as it would not influence the final result of the system.

## Conclusions

This paper proposes a system for detecting the occupancy of a parking lot which uses computer vision techniques and machine learning. The most commonly used techniques in the literature only make use of computer vision and image classification to determine the occupancy status of a parking space. There are few previous works in this field that make use of machine learning to identify the vehicles present in a parking lot with the purpose of determining occupancy. None of them propose a system with the characteristics of the solution presented in this paper, that is to say one that considers occupancy according to the area of occupation of each space.

To analyze the correctness of the algorithm and verify the viability of the system, multiple scenarios have been designed to test the precision of the modules that comprise it. First, the detection of parking spaces is analyzed, something that was not done in previous work but which is necessary for deployment in real environments, obtaining an accuracy of 95.90%. Then, the precision in the detection of vehicles using computer vision and artificial intelligence is analyzed, obtaining an accuracy of 98.63% in synthetic environments and 97.98 % in real environments. Finally, the accuracy of the algorithm that identifies and locates the vehicles in the parking lot and determines the occupancy of each of the spaces, and therefore of the system, is 98.21%.

With these results, it can be affirmed that the approach of a vehicle area-based detection system that seeks to optimise parking spaces to the maximum is possible and valid. The proposed system is capable of obtaining very good accuracy, with results above 98% in the simulated environment and close to 98% in real environments.

However, the system has limitations in that the images must be taken for the detection to work properly. These images must be taken from an elevated position to obtain a zenithal view of the parking spaces to be analysed, which makes it difficult to use in indoor car parks. n addition, the time required for the processing of each image of 1.5s, although acceptable for managing a car park in real time, is an aspect to be improved in the following iterations of the system. In addition to this, the following evolutions will be oriented towards the use of the system in real environments. To this end, the datasets will be improved to cover a greater number of cases, the processing time of each of the images will be optimised and the performance will be evaluated by analysing videos of car parks.

This work aims to open a new line of research in the area of parking management systems, aligned with technological advances in computer vision and artificial intelligence, seeking to create more intelligent systems capable of detecting the exact position and size of vehicles, improving the optimisation of parking spaces.
